# TBK1, a prioritized drug repurposing target for amyotrophic lateral sclerosis: evidence from druggable genome Mendelian randomization and pharmacological verification in vitro

**DOI:** 10.1186/s12916-024-03314-1

**Published:** 2024-03-05

**Authors:** Qing-Qing Duan, Han Wang, Wei-Ming Su, Xiao-Jing Gu, Xiao-Fei Shen, Zheng Jiang, Yan-Ling Ren, Bei Cao, Guo-Bo Li, Yi Wang, Yong-Ping Chen

**Affiliations:** 1 Department of Neurology, West China Hospital, Sichuan University, Chengdu, Sichuan China; 2 Institute of Brain Science and Brain-Inspired Technology, West China Hospital, Sichuan University, Sichuan, Chengdu,, 610041 China; 3 Rare Disease Center, West China Hospital, Sichuan University, Sichuan, Chengdu, 610041 China; 4 Department of Pathophysiology, West China College of Basic Medical Sciences and Forensic Medicine, Sichuan University, Sichuan, Chengdu, 610041 China; 5 Mental Health Center, West China Hospital, Sichuan University, Sichuan, Chengdu, 610041 China; 6 TCM Regulating Metabolic Diseases Key Laboratory of Sichuan Province, Hospital of Chengdu University of Traditional Chinese Medicine, Chengdu University of Traditional Chinese Medicine, Chengdu, 610072 China; 7 Key Laboratory of Drug Targeting and Drug Delivery System of Ministry of Education, West China School of Pharmacy, Sichuan University, Chengdu, 610041 China

**Keywords:** Amyotrophic lateral sclerosis, Mendelian randomization, Drug repurposing, Druggable gene, TBK1

## Abstract

**Background:**

There is a lack of effective therapeutic strategies for amyotrophic lateral sclerosis (ALS); therefore, drug repurposing might provide a rapid approach to meet the urgent need for treatment.

**Methods:**

To identify therapeutic targets associated with ALS, we conducted Mendelian randomization (MR) analysis and colocalization analysis using cis-eQTL of druggable gene and ALS GWAS data collections to determine annotated druggable gene targets that exhibited significant associations with ALS. By subsequent repurposing drug discovery coupled with inclusion criteria selection, we identified several drug candidates corresponding to their druggable gene targets that have been genetically validated. The pharmacological assays were then conducted to further assess the efficacy of genetics-supported repurposed drugs for potential ALS therapy in various cellular models.

**Results:**

Through MR analysis, we identified potential ALS druggable genes in the blood, including *TBK1* [OR 1.30, 95%CI (1.19, 1.42)], *TNFSF12* [OR 1.36, 95%CI (1.19, 1.56)], *GPX3* [OR 1.28, 95%CI (1.15, 1.43)], *TNFSF13* [OR 0.45, 95%CI (0.32, 0.64)], and *CD68* [OR 0.38, 95%CI (0.24, 0.58)]. Additionally, we identified potential ALS druggable genes in the brain, including *RESP18* [OR 1.11, 95%CI (1.07, 1.16)], *GPX3* [OR 0.57, 95%CI (0.48, 0.68)], *GDF9* [OR 0.77, 95%CI (0.67, 0.88)], and *PTPRN* [OR 0.17, 95%CI (0.08, 0.34)]. Among them, *TBK1*, *TNFSF12*, *RESP18*, and *GPX3* were confirmed in further colocalization analysis. We identified five drugs with repurposing opportunities targeting *TBK1*, *TNFSF12*, and *GPX3*, namely fostamatinib (R788), amlexanox (AMX), BIIB-023, RG-7212, and glutathione as potential repurposing drugs. R788 and AMX were prioritized due to their genetic supports, safety profiles, and cost-effectiveness evaluation. Further pharmacological analysis revealed that R788 and AMX mitigated neuroinflammation in ALS cell models characterized by overly active cGAS/STING signaling that was induced by MSA-2 or ALS-related toxic proteins (TDP-43 and SOD1), through the inhibition of TBK1 phosphorylation.

**Conclusions:**

Our MR analyses provided genetic evidence supporting *TBK1*, *TNFSF12*, *RESP18*, and *GPX3* as druggable genes for ALS treatment. Among the drug candidates targeting the above genes with repurposing opportunities, FDA-approved drug-R788 and AMX served as effective TBK1 inhibitors. The subsequent pharmacological studies validated the potential of R788 and AMX for treating specific ALS subtypes through the inhibition of TBK1 phosphorylation.

**Supplementary Information:**

The online version contains supplementary material available at 10.1186/s12916-024-03314-1.

## Background

Amyotrophic lateral sclerosis (ALS) is a fatal motor neuron disease characterized by progressive degeneration of nerve cells in the spinal cord and brain, leading to disability and eventual death of patients from respiratory failure. The prevalence of ALS is about 5 in 100,000, while patients usually die within 2 to 5 years of onset [1]. Due to a lack of understanding in its pathogenesis, formidable challenges have been historically posed on therapeutic approaches. Currently, riluzole, edaravone, AMX0035, and tofersen have been approved by FDA for treating ALS by reducing glutamate release, acting as an antioxidant, mitigating mitochondrial dysfunction, as well as down-regulating superoxide dismutase 1 (SOD1) protein level, respectively. However, most of them were demonstrated to slightly slow down ALS progression by a couple of months [2, 3]. Therefore, it is a priority to develop more efficient chemical compounds for urgent need to treat this devastating disease.

Accurately identifying drug candidates for particular disease subtypes and verifying their impacts on disease progression are prerequisites in drug development. However, due to the small sample size of the trial cohort, genetic complexity and heterogeneity, and insufficient drug efficacy or safety data, most clinical trials for newly developed drugs have to be terminated with unsatisfactory results [4]. Meanwhile, several studies have reported that the genetically supported drug-repurposing strategies could double the success rates in clinical development [5], and numerous large-scale genome-wide association studies (GWASs) have identified many single-nucleotide polymorphisms (SNPs) associated with ALS risk, offering possibilities for shortening the innovation period of ALS drug. According to Mendel’s law of independent segregation, genetic variants are randomly allocated to individuals during gamete formation and fertilization. Mendelian randomization (MR) applies these genetic variants (instrumental variables, IVs) to study causality with less confounding or unbias than that in traditional epidemiological methods. Therefore, MR has been considered an ingenious method to harness high power and fidelity of randomization in human biomedical research [6, 7]. The druggable genes, as described by Finan et al. [8], refers to the selection of genes that have the potential to serve as targets for pharmacological intervention. In drug-target MR analysis, cis-expression quantitative trait loci (*cis*-eQTL) located in the genomic region of the drug-target gene are often considered proxies, which have been intensively utilized to analyze the regulatory relationship between nucleotide variants and gene expression fluctuations [9, 10]. Such MR analyses have been applied to multiple diseases, such as Parkinson’s disease (PD) [11] and Alzheimer’s disease (AD) [12].

ALS GWAS have revealed numerous genes that exhibit statistical associations with the disease [13]. Among them, *TNFSF12,* also called *TWEAK*, has been researched in tumors, autoimmune disease, and inflammatory diseases [14–18]. Studies have shown that *TNFSF12* plays a crucial role as an apoptosis inducer in the pathogenesis of muscle atrophy and is associated with neuroinflammation in multiple sclerosis [19, 20]. GPX3 is abundantly present in neurons, the brain, and other tissues [21]. It is a well-known glutathione peroxidase, performing antioxidant functions associated with well-recognized ALS gene like *SOD1* [22, 23]. *TBK1* gene encoding TANK-binding kinase-1 (TBK1) has been shown to closely interplay with ALS development. Multiple loss-of-function mutations on *TBK1* gene have been identified across sporadic and familial instances of ALS, as well as in cases of ALS/frontotemporal dementia (ALS-FTD/FTD) [24]. On the other hand, within ALS subtypes linked to gain-of-function mutations of SOD1 or TARDBP (encoding TAR DNA-binding protein, TDP-43), it has been observed that mitochondrial damage caused by mutated proteins could potentially stimulate the release of mitochondrial DNA, thereby activating the cyclic GMP-AMP synthase (cGAS)/stimulator of interferon genes (STING) pathway to potently initiate or aggravate neuroinflammation [25, 26]. Nowadays, extensive cellular and animal studies have demonstrated the protective effects of chemical inhibitors targeting upper stream factors like STING and cGAS in ALS models, while breakthroughs in clinical application remain to be achieved [25, 27].

In the current study, we obtained eQTL data for the druggable genome and conducted MR with the outcome of ALS GWAS to identify potential druggable genes associated with ALS. Through further colocalization analysis, we obtained the target genes with the stronger genetic association, including *TBK1*, *TNFSF12*, *RESP18*, and *GPX3*. Subsequently, we delved into ALS-repurposed drugs related to druggable genes through a series of criteria and identified R788 and AMX. Moreover, we conducted further research into druggable gene targets and the pharmacological effects of their targeted drugs in the context of ALS cell models. Lastly, AMX and R788 were verified to hold the therapeutic potential in attenuating ALS progression via dampened neuroinflammation.

## Methods

**Fig. 1:**
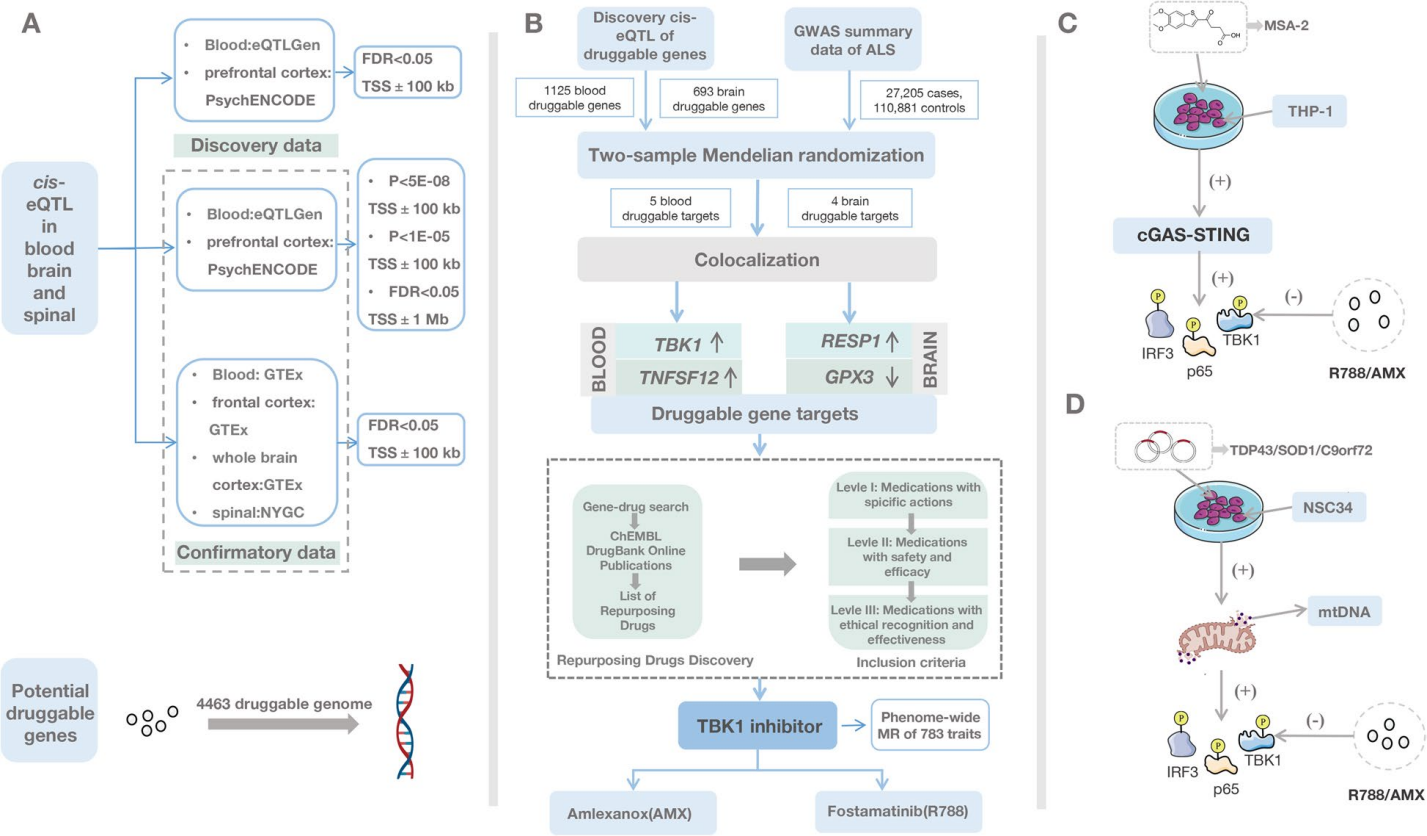
Graphical abstract of genetic instrument variants selection, Mendelian randomization, identification of repurposing drugs discovery, and pharmacological analysis. **A** Obtaining cis-eQTL data for druggable genes by overlapping discovery cis-eQTL data and confirmatory cis-eQTL data with potentially druggable genes. The SNP data obtained through filtering for cis-eQTL data for blood and brain tissue from eQTLGen and PsychENCODE, with located within ± 100 kb of the TSS and meeting the FDR < 0.05 criteria, is considered as “Discovery cis-eQTL data”. The different sets of SNPs based on various additional selection criteria and multiple eQTL datasets are considered as “Confirmatory cis-eQTL data.” **B** Workflow showing the MR study and repurposing drugs discovery. Identifying druggable genes with strong genetic associations through the MR and colocalization analysis, further exploring treatment drugs associated with these druggable genes that meet the inclusion criteria. **C** R788 and AMX inhibit MSA-2-induced cGAS-STING signaling activation. **D** R788 and AMX inhibit ALS-related toxic protein mediated cGAS/STING signaling. MR Mendelian randomization, *cis*-eQTL *cis*-expression quantitative trait loci, ALS amyotrophic lateral sclerosis

All DNA positions are based on the human reference genome build hg19 (GRCh37). Data processing was completed using R software version 4.1.1. The main R package involved in this study included “TwoSampleMR”, “dplyr”, “ieugwasr”, “org.Hs.eg.db “, “Homo. sapiens”, and “coloc”. This MR study was in accordance with the guidelines of Strengthening the Reporting of Observational Studies in Epidemiology using Mendelian Randomization (STROBE-MR) [28, 29]. The checklist is elaborated in detail in Additional file 1: Table S1. Flowchart of our study is shown in Fig. 1A-D.

### Potential druggable genes list

The set of genes comprising the druggable genome was obtained from a recent review [8], which estimated 4479 of the 20,300 protein-coding genes. A total of 4463 unique gene symbols were identified as druggable genes, and 16 pseudogenes were absent.

### eQTLs data

In primary MR analysis, we searched and downloaded cis-eQTL data for blood and brain tissue from eQTLGen (www.eqtlgen.org) and PsychENCODE (http://resource.psychencode.org). eQTLGen provides *cis*-eQTL data consisting of 31,684 blood and peripheral blood mononuclear cell samples, and most individuals were of European ancestry. We downloaded the full statistically significant *cis*-eQTLs results (FDR < 0.05) updated in 2019, in which each *cis*-eQTL had been validated in at least two cohorts [30]. Meanwhile, we obtained the allele frequencies from eQTLGen which were calculated using reported allele counts from all cohorts.

For brain tissue, we obtained the full significant *cis*-eQTLs from PsychENCODE, which performed multiple testing by limiting FDR values to less than 0.05 and requiring genes to have an expression > 0.1 FPKM in at least ten samples [31]. The *cis*-eQTL data of brain tissue was performed by genotype and gene expression data from 1387 individuals, including 679 healthy controls, 497 schizophrenia, 172 bipolar disorder, 31 autism spectrum disorder, and eight affective disorder patients.

In order to investigate potential horizontal pleiotropy and eliminate confounding factors, we utilized PhenoScanner [32] (http://www.phenoscanner. medschl.cam.ac.uk/) to search for all eligible SNPs, excluding confounders related to ALS (such as obesity, BMI, and diabetes) [33–35] and SNPs associated with ALS. In addition, to investigate whether the IVs corresponding to causal genes identified via the blood and brain MR and colocalization have an impact on the expression of other genes, we utilized data from the GTEx project (https://www.gtexportal.org/home/datasets).

We defined the eQTL data from both blood and brain tissues mentioned above as “Discovery eQTL data.” To enhance the reliability of our MR results, we further validated them using different databases from the GTEx version 8 dataset and NYGC ALS Consortium [36], referred to as “Confirmatory eQTL data.” More details are provided in the Additional file 2 and all the details of the eQTL data are presented in Additional file 1: Table S2.

### ALS GWAS data

The outcome GWAS was performed in 29,612 patients with ALS and 122,656 controls; we used the summary results conducted in European ancestry populations (27,205 cases, 110,881 controls) [13]. Full data descriptions are available in the original publications [13].

### Mendelian randomization

The *cis*-eQTLs of druggable genes were used as IVs to identify new drug targets in the MR analysis. Firstly, we obtained the gene symbol for all genes and filtered out pseudogenes. Secondly, we matched the transcription start site (TSS) location for each gene symbol and only included the SNPs within the ± 100 kb from TSS and the FDR < 0.05 [30]. In order to enhance result confidence, we established various SNP selection criteria and selected multiple eQTL datasets, the details can be found in the Additional file 2. Thirdly, we overlap the remaining *cis*-eQTLs in blood or brain tissue and the druggable gene symbols list. SNPs were then clumped at *r*^2^ < 0.001 using European samples from the 1000 Genomes Project in each *cis*-QTL as the IVs for MR. Lastly, we performed MR analyses using the package “TwoSampleMR” (https://mrcieu.github.io/TwoSampleMR/) via R version 4.1.1. We used inverse-variance weighted (IVW) MR for IVs that contain more than one variant and Wald ratio for only one IV. When there are more than two variants included in the gene IVs, we conducted a sensitivity analysis using the MR-Egger and weighted median methods. We identified significant MR results using an FDR value threshold of FDR < 0.05. In addition, the Steiger test was used to verify the orientation.

### Colocalization

Colocalization analysis was performed between each significant blood and brain gene’s eQTL and ALS outcome using the “coloc” package (https://github.com/clagiamba/moloc). We used default priors: *P*_1_ = 10^−4^, *P*_2_ = 10^−4^, *P*_12_ = 10^−5^. *P*_1_, *P*_2,_ and *P*_12_ are the prior probabilities that a SNP in the tested region is significantly associated with the expression of the tested gene, the tested ALS outcome, or both, respectively. It calculates the posterior probabilities of five optional assumptions: H0, not associated with any trait; H1, related to trait 1 but not to trait 2; H2, related to trait 2 but not to trait 1; H3, two independent correlations signals, which do not correlate with each other; H4, the common correlation signal of two traits. Because the H4 modeling assumption indicates that both traits are driven by the same causal variant, we prefer that the H4 assumption holds. A PP4 > 0.75 threshold was set to filter comparisons with high support for an association with both traits [37].

### Repurposing drug discovery and inclusion criteria

For the potential druggable genes that exhibited significant colocalization with ALS GWAS signals in MR analysis, we conducted a gene-drug search using the ChEMBL (www.ebi.ac.uk), DrugBank Online (go.drugbank.com) databases, and previous publications to identify potential repurposing drugs that could act on druggable gene targets from MR and colocalization analysis. The obtained candidates then went through three inclusion criteria filtration: Level I: Drugs with specific actions; Level II: Drugs with safety and efficacy; and Level III: Drugs with ethical approval and proven therapeutic effectiveness. Among them, drugs passed all three levels of selection were prioritized for drug repurposing tests.

### Phenome-wide MR

To predict drug safety and risk for on-target adverse effects, phenome-wide MR analysis was conducted to systematically infer the causal effects of the expression of prior druggable genes on 783 non-ALS disease traits in the European ancestry population from the UK Biobank [38]. Summary statistics of disease-associated SNPs were downloaded from the SAIGE GWAS (https://www.leelabsg.org/resources).

### Reagents and antibodies

Products from the following vendors were used as reagents: MSA-2 (MedChemExpress, catalog no. HY-136927); phorbol-12-myristate-13-acetate (PMA) (Beyotime, catalog no. S1819); fostamatinib (MedChemExpress, catalog no. HY-13038A); and amlexanox (MedChemExpress, catalog no. HY-B0713). In addition, antibodies from the following manufacturers were used: TBK1 (Beyotime, catalog no. AF8103); p-TBK1 (Cell Signaling Technology, catalog no. 5483 T); p-p65 (Cell Signaling Technology, catalog no. 3303 T); p-IRF3 (Cell Signaling Technology, catalog no. 29047S); TDP-43 (HUABIO, catalog no. ET1703-74); SOD1 (HUABIO, catalog no. ET1702-36); β-actin (Cell Signaling Technology, catalog no. 3700S); STING (Proteintech, catalog no. 19851–1-AP); cGAS (HUABIO, catalog no. HA500023); p-SYK (Cell Signaling Technology, catalog no. 2710S); Flag (Beyotime, catalog no. AF2852); IKK epsilon (Zenbio, catalog no. R382375); IRF3 (HUABIO, catalog no. ET1612-14); and NF-κB p65 (HUABIO, catalog no. ET1603-12).

### Cell culture and treatments

The growth medium for mouse neuronal cell line NSC-34 cells was composed of Dulbecco’s modified Eagle’s medium (DMEM) (Gibco, catalog no. 11965092) supplemented with 10% fetal bovine serum (FBS) (Gibco, catalog no. 10270–106) and 1% penicillin–streptomycin (PS) (Gibco, catalog no. 15140–122). For cultivating THP-1 cells, we used RPMI-1640 medium containing 10% FBS, 1% PS, and 0.05 mM β-mercaptoethanol.

THP-1 cells underwent initial treatment with 100 ng/mL PMA for 48 h, facilitating their transformation into adherent macrophages. Subsequently, these cells were pre-treated with either 10-20 μM R788 or 100-200 μM AMX for 4 h, prior to stimulation with 20 μM MSA-2 for 1 h, which elicited p-TBK1 expression. After that, protein expression levels were evaluated employing Western blotting. However, the drug treatment method was slightly modified for cells undergoing RT-qPCR experiments. Cells were initially pre-treated with either R788 or AMX for 1 h, followed by a 6-h induction with MSA-2.

### Establishment of stable cell lines through lentiviral transduction

Third-generation lentiviral constructs, encompassing pLVX-TRE3G-teton-puro (vector), human TDP-43^WT^, and its mutant TDP43^Q331K^, as well as human SOD1^WT^ and its mutant SOD1^G93A^, were utilized for lentivirus production, following established procedures. HEK293T cells underwent transient transfection with the target plasmid, pAX plasmid, and pMD plasmid, employing PEI diluted in serum-free DMEM medium to generate lentiviral particles. After a 48-h incubation, the cell culture supernatant was harvested and centrifuged at 4000 rpm, at 4 °C, for 4 min. NSC-34 cells were exposed to lentiviral particles bearing either GFP as a control or the target plasmid in the presence of polybrene (1000 ×). Post 48-h infection, transduced cells were subjected to antibiotic selection using puromycin, facilitating the creation of stable cell lines featuring doxycycline-inducible 3 × FLAG-tagged TDP-43 or 1 × FLAG-tagged SOD1.

### Plasmid constructs and transfection

Site-directed mutagenesis was conducted to obtain pLenti-TRE-gene-CBH-Tet-On@3G-Flag-TARDBP Q331K using the Q5 Site-Directed Mutagenesis Kit (New England Biolabs, catalog no. E0554) and specifically designed primers (Additional file 1: Table S3). The successful introduction of point mutations was confirmed via Sanger sequencing.

By the manufacturer’s guidelines for transfection reagents, target plasmids were introduced into NSC-34 cells using Lipofectamine 3000 (Life Technologies) for 48 h. Cells were subsequently collected for Western blotting and RT-qPCR analyses.

### Cytotoxicity test: CCK-8 assay

By the manufacturer’s instructions, cell toxicity was evaluated using the Cell Counting Kit-8 (Cellcook Biotech, catalog no. CT01A). THP-1 cells were initially seeded in a 96-well plate and subjected to 100 ng/mL PMA treatment for 48 h, facilitating their transformation into adherent macrophages. Next, cells were treated with either 50–200 μM AMX or 1.25–20 μM R788 for 5 h, employing DMSO as a negative control. Subsequently, 10 μL of CCK-8 solution was added to each well, with a 1-h incubation period at 37 °C. Ultimately, a microplate reader (Thermo Fisher Scientific) was utilized to measure cell absorbance at 450 nm.

### Western blotting

Total protein extracts designated for Western blotting analysis were obtained from cell homogenates using RIPA Lysis and Extraction Buffer (Thermo Scientific, catalog no. 89900), supplemented with freshly added protease and phosphatase inhibitor single-use cocktails (Thermo Scientific, catalog no. 1861280). Equal concentrations of each protein sample were subjected to 8–12% sodium dodecyl sulfate–polyacrylamide gel electrophoresis (SDS-PAGE). The separated proteins were then transferred onto polyvinylidene difluoride (PVDF) membranes and blocked for 20 min using a protein-free rapid-blocking buffer (Shanghai Little Jumping Frog Biotechnology, catalog no. BD307L). The membranes were incubated overnight at 4 °C with primary antibodies and subsequently washed five times with Tris-buffered saline containing 0.1% Tween 20 detergent (TBST). Following this, membranes were incubated for 1 h with horseradish peroxidase-conjugated secondary antibodies (Beyotime, catalog no. A0216), (Beyotime, catalog no. A0208). After an additional five 10-min washes with TBST, protein bands were detected using the ChemiDoc Touch Imaging System (BioRad).

### Quantitative real-time PCR

In accordance with the manufacturer’s instructions, total RNA from cells was isolated using TRIzol Reagent (Ambion, catalog no. 15596026), and the extracted RNA was reverse-transcribed into cDNA using the HiScript III All-in-one RT SuperMix for qPCR (Vazyme, catalog no. R333). Following the manufacturer’s protocol, quantitative real-time PCR was performed on a QuantStudio 7 Real-Time PCR System (Thermo Fisher Scientific) with ChamQ SYBR qPCR Master Mix (Vazyme, catalog no. R311). GAPDH served as an internal control, and the 2^−ΔΔCt^ method was employed to analyze relative changes in gene expression. All primer sequences used in this study can be found in Additional file 1: Table S3.

### Quantification and statistical analysis

All experiments were executed with at least three technical replicates, and the data are generally expressed as the mean ± SEM. For statistical evaluation, either Student’s *t*-test (2 groups) or one-way or two-way ANOVA was employed, followed by subsequent analysis using GraphPad Prism 9. Significance thresholds are indicated as: *p < 0.05; **p < 0.01; ***p < 0.001; and ns (not significant).

## Results

### Genetic instrument variants selection

After overlapping the significant *cis-*eQTL and the list of druggable gene symbols, we obtained 2347 blood-derived and 1679 brain-derived druggable genes (Fig. 1A). According to the selection criterion of instrument variants, in discovery analysis, a total of 1601 SNPs for 1125 blood-druggable genes and 693 SNPs for 648 brain-druggable genes were used as IVs for further MR analysis (Additional file 1: Table S4-5). To enhance result confidence from the discovery analysis, the different sets of SNPs as IVs based on the various additional selection criteria and multiple eQTL datasets were shown in the Additional file 3.

### MR analysis between gene expression and ALS outcomes

Using a significance threshold of SNPs located within ± 100 kb of the TSS and meeting the FDR < 0.05, we found eight unique potential druggable targets (*TNFSF13*, *CD68*, *TNFSF12*, *TBK1*, *RESP18*, *GDF9*, *PTPRN*, and *GPX3*) in the blood and brain (Fig. 1B “Mendelian randomization” section). Particularly in the blood, MR analysis results showed that *TNFSF13* and *CD68* were associated with reduced ALS risk (*TNFSF13*: OR 0.45, 95% CI 0.32–0.64; *CD68*: OR 0.38, 95% CI 0.24–0.58), while *TNFSF12*, *TBK1*, and *GPX3* were associated with increased ALS risk (*TNFSF12*: OR 1.36, 95% CI 1.19–1.56; *TBK1*: OR 1.30, 95% CI 1.19–1.42; *GPX3*: OR 1.28, 95% CI 1.15–1.43) (Additional file 1: Table S4).

In the brain, *RESP18* expression was associated with a higher risk of ALS (OR 1.30; 95% CI 1.19–1.42), while *GDF9*, *PTPRN*, and *GPX3* were associated with lower risks of ALS (*GDF9*: OR 0.77, 95% CI 0.67–0.88; *PTRRN*: OR 0.17, 95% CI 0.08–0.34; *GPX3*: OR 0.57, 95% CI 0.48–0.68) (Additional file 1: Table S5).

**Fig. 2:**
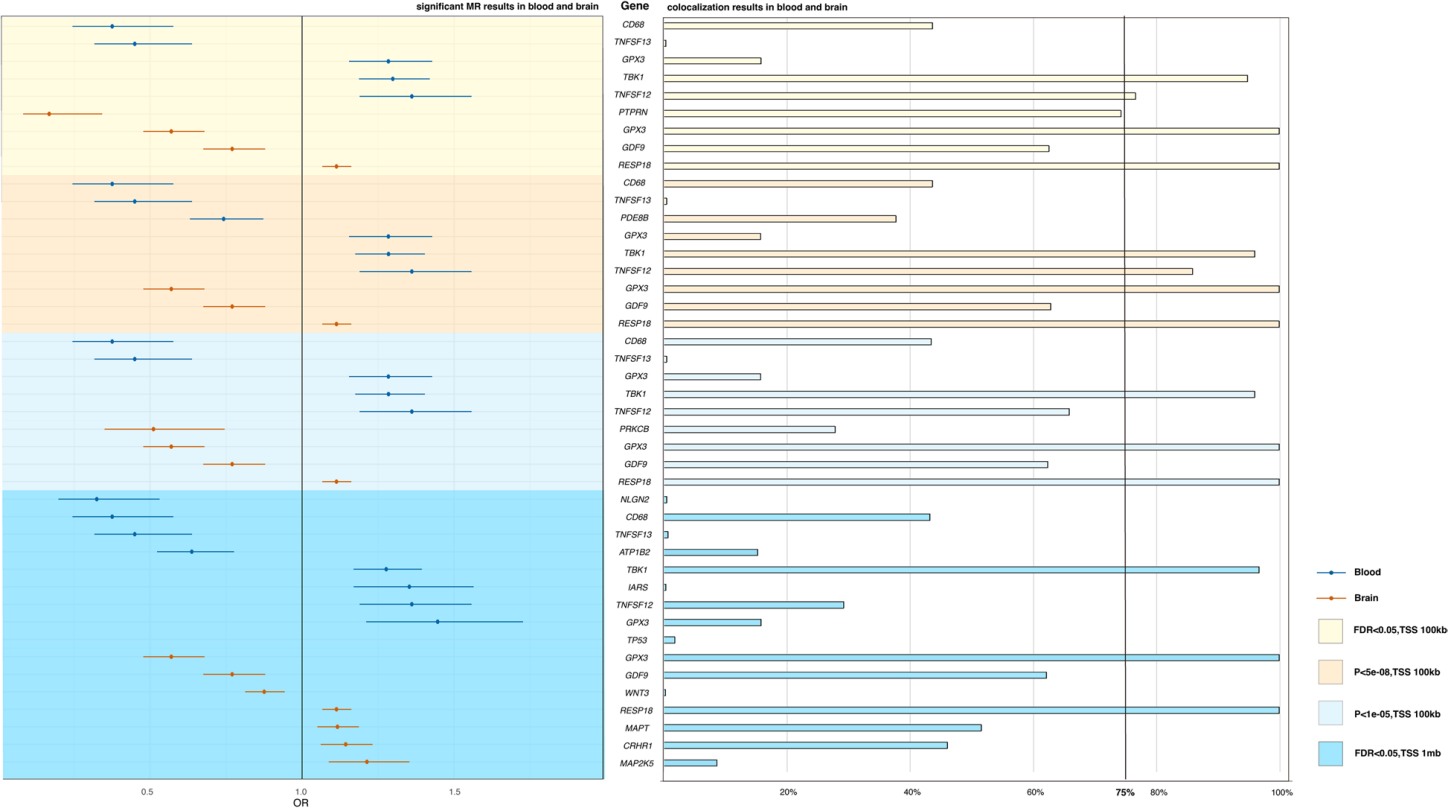
MR and colocalization results of potentially druggable genes. The forest plot displays the results of potential druggable genes under different MR parameters. The bar charts illustrate the colocalization results for each potential druggable gene. Results are color-coded according to tissue (blue = blood, red = brain tissue). FDR false discovery rate, OR odds ratio

Most importantly, we validated the above results using different SNP selection parameters and additional blood, brain, and spinal databases, confirming the roles of *TBK1*, *TNFSF12*, *RESP18*, and *GPX3* in ALS (Additional file 1: Table S4-6, Additional file 3). We summarized the list of the potentially druggable genes (Additional file 1: Table S7) and drew figures based on the MR of these genes (Fig. 2).

The PhenoScanner’s results revealed that 15 blood SNPs (rs4075482, rs11030014, rs115799091, rs11642612, rs12961563, rs1475974, rs17771137, rs34587452, rs3772704, rs4705849, rs57311037, rs6060266, rs6494672, rs6544039, and rs9487064) and 11 brain SNPs (rs112366001, rs1131017, rs116678101, rs236650, rs6457788, rs677, rs74400203, rs7557796, rs79113395, rs823114, and rs942650) were correlated with confounding factors. After excluding these SNPs and reapplying MR analysis, the list of potential ALS druggable genes remained unchanged.

By analyzing in GTEx whether the IVs corresponding to causal genes have an impact on the expression of other genes, we did not observe any effects of *TBK1*’s IV on other genes. However, IV associated with the *TNFSF12* was found to influence the expression of multiple genes, including *ATP1B2*, *CHRNB1*, *EIF4A1, FGF11*, *POLR2A*, *SAT2*, *SENP3*, *TNFSF13*, and *ZBTB4*. Additionally, the IV of *GPX3* was discovered to affect the expression of *TNIP1*, while IV related to *RESP18* was able to influence the expression of *AC053503.12*, *AC053503.4*, *GLB1L*, *PTPRN*, and *ZFAND2B*.

Additionally, the MR analysis results show that gene IVs containing more than 2 SNPs exist only in the blood (data from eQTLGen), and we conducted sensitivity analysis on these genes. Sensitivity analysis results indicate that all genes with more than 2 SNPs passed the tests in MR-Egger and weighted median methods, except for *STK32C*, *PXDN*, *PAM*, *CD93*, and *SIRPB1* (*P* < 0.05) (Additional file 1: Table S8).

### Colocalization result

We performed colocalization analysis of significant genes obtained from the MR analysis and ALS GWAS signals (Fig. 1B “Colocalization” section) and then found a list of genes which was colocalized with the GWAS variants with high support (PP_4_ > 0.75). There is determined colocalization for two significant genes in the blood (*TBK1* and *TNFSF12*) and two in the brain (*RESP18* and *GPX3*) (Fig. 2). The results of colocalization analysis for all eight potential druggable genes are summarized in Additional file 1: Table S9-10.

### Discovery of repurposed drugs

To further identify potential repurposing opportunities to informed trials of ALS patients, we searched ChEMBL, DrugBank Online, and previous publication [39]. As a result, we obtained five drugs acting on three ALS-related gene targets, including *TBK1*, *TNFSF12*, and *GPX3*, respectively (Table 1). Based on our inclusion criteria, the candidate drugs were categorized into three strata: glutathione is a tripeptide that participates in various physiological and pathological conditions [40]. While glutathione may antagonize the neuro-oxidative stress response caused by GPX3 deficiency [41], we excluded this chemical from the primary selection (Level I) due to its broad spectrum of action. BIIB023 and RG7212 are both anti-TNFSF12 antibodies to potentially regulate inflammatory processes in neurons and tumor tissues [42, 43], with ongoing clinical investigations (ClinicalTrials.gov identifier NCT01407406 for BIIB023 and NCT01383733 for RG7212). Due to the uncertain safety and efficacy, these two biologics failed to pass the Level II selection. AMX is an FDA-approved TBK1 inhibitor [39], while R788 is a FDA-approved SYK inhibitor showing high affinity with TBK1 [44]. They were considered to meet the ethical recognition and effectiveness, which made them pass the Level III selection (Fig. 1B "Repurposing Drugs discovery" and "inclusion criteria" sections).

**Table 1:** Three druggable gene targets of ALS and five existing drugs identified for drugs repurposing application. Tier 1 contains the objectives of the approved drug and clinical drug, tier 2 contains incorporated proteins closely related to drug targets or with associated drug-like compounds, and tier 3 contains incorporated extracellular proteins and members of key drug-target families [8]. All information on medications is from ChEMBL and DrugBank Online

Gene	Target	Tier of druggable genes	Tissue	Drug name	Target ChEMBL ID	Action type	Max clinical phase	Indication
*TBK1*	TANK-binding kinase 1	Tier 1	Blood	Fostamatinib	CHEMBL2103830	Inhibitor	Approved	Immune thrombocytopenic purpura
				Amlexanox	NA	Inhibitor	Approved	Aphthous ulcers
*TNFSF12*	Tumor necrosis factor (ligand) superfamily, member 12	Tier 1	Blood	BIIB-023	CHEMBL2109598	Antibody	2	Lupus nephritis; rheumatoid arthritis
				RG-7212	CHEMBL2109600	Antibody	1	Neoplasms
*GPX3*	Glutathione peroxidase 3 (plasma)	Tier 1	Blood and brain	Glutathione	CHEMBL1543	Cofactor	3	Cystic fibrosis; reperfusion injury; pancreatic neoplasms; Parkinson disease; breast neoplasms; pain; peripheral nervous system diseases; Persian Gulf Syndrome

### Identification of AMX and R788 as promising inhibitors targeting TBK1

It has been published that AMX binds to the hinge region of TBK1 to play a crucial role in mediating the enzymatic activities of TBK1 and IKKε (Additional file 4: Fig S1A, S1B) [45, 46]. We employed a cellular thermal shift assay (CETSA) to investigate the impact of these two chemicals on TBK1 thermal stability. The result showed that in comparison to the vehicle control, the influence of AMX on the thermal stability of TBK1 in NSC-34 cell lysates exhibited moderate differences within the temperature range of 37–60 °C (Additional file 4: Fig S1C).

**Fig. 3:**
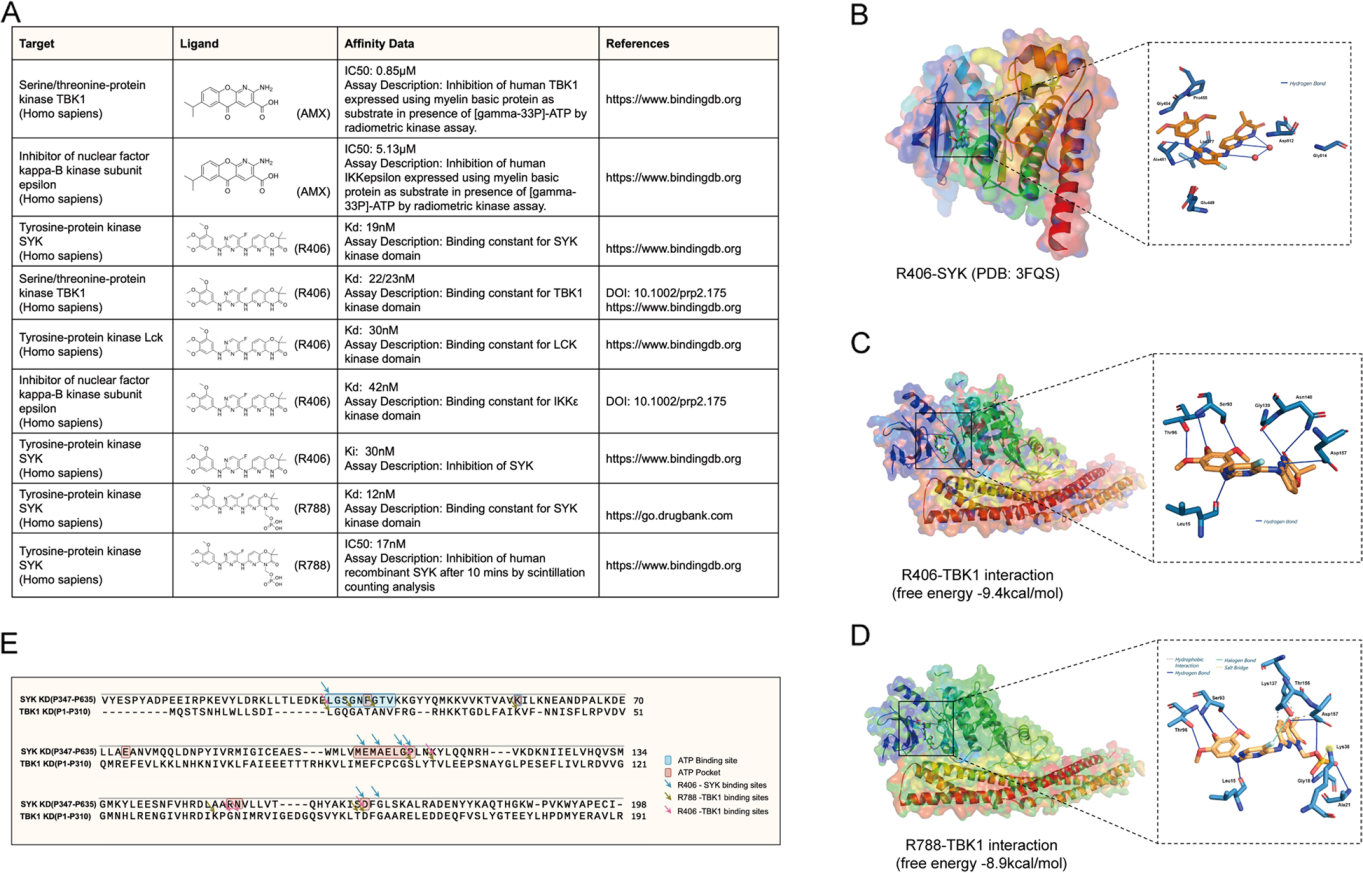
TBK1 was identified as the binding target of FDA-approved drugs. **A** A summary of the published TBK1 binding affinity measurements of AMX, R406, and R788. **B** The global (left) and local (right) schematics illustrate the interaction between R406 and SYK by co-crystal structure (PDB: 3FQS) [48]. **C** Molecular docking model showing the global (left) and local (right) interactions between R406 and TBK1 (PDB: 4IM0) (predicted free energy: − 9.4 kcal/mol) (hydrogen bonds were depicted by solid blue line). **D** Molecular docking model showing the global (left) and local (right) interactions between R788 and TBK1 (PDB: 4IM0) (predicted free energy: − 8.9 kcal/mol) (hydrogen bonds were depicted by solid blue line, hydrophobic interactions were depicted by black dash lines, halogen bonds were depicted by solid green lines, and salt bridges were depicted by yellow dash lines). **E** The amino acid sequence comparison between SYK and TBK1 kinase domains

Known as a SYK inhibitor, R788 and its active compound R406 have been confirmed to interact with a range of protein kinase targets, including TBK1 by multiple pharmacological analyses [47]. Online databases including Lincs (https://lincs.hms.harvard.edu), DrugBank, and BindingDB (https://www.bindingdb.org/rwd/bind/index.jsp) demonstrated R406 possess a strong affinity to TBK1 (Kd =  ~ 22 nM), which was comparable to SYK binding affinity (Kd =  ~ 19 nM) (Fig. 3A). The published co-crystal structure revealed that R406 was embedded within the SYK kinase domain and occupied the ATP-binding pocket. It formed two hydrogen bonds with hinge residue Ala451 and aromatic CH-O interactions with the Glu449 backbone carbonyl group (Fig. 3B) [48]. Notably, all above drug-interacting residues shared similarities in the key amino acids presented in the ATP pockets and ATP-binding regions of TBK1.

R788 undergoes rapid conversion to R406 in vivo [47], while its metabolic process in cellular experiments is unknown. Therefore, we conducted separate molecular docking simulations to investigate the interactions between TBK1 and each chemical. Our data showed both chemicals well fit into TBK1 kinase domain, where the free energies were − 9.4 kcal/mol and − 8.9 kcal/mol, respectively (Fig. 3C–D). Notably, R406 formed hydrophobic interactions with six amino acid residues of TBK1, five of which (Ser93, Leu15, Gly139, Asn140, Asp157) were found to be located within the annotated ATP-binding site or ATP-pocket (Fig. 3E). On the other hand, the predicted R788-TBK1 interactions shows hydrogen bonds (with Ser93, Thr96, Leu15, Gly18, Ala21, Asp157, Thr156, Lys137), hydrophobic interactions (with Lys137, Asp157), halogen bond (with Thr156), and salt bridge (with Lys38). Among them, six amino acids (Ser93, Leu15, Gly18, Ala21, Lys38, Asp157) were located in the same motif (Fig. 3E). These findings indicated the high interacting affinities between TBK1 and R788 or R406, albeit no obvious thermal stability change of TBK1 upon R788 administration was seen in the CETSA assay (Additional file 4: Fig S1D).

### R788 and AMX inhibited MSA-2-induced cGAS/STING activation

**Fig. 4:**
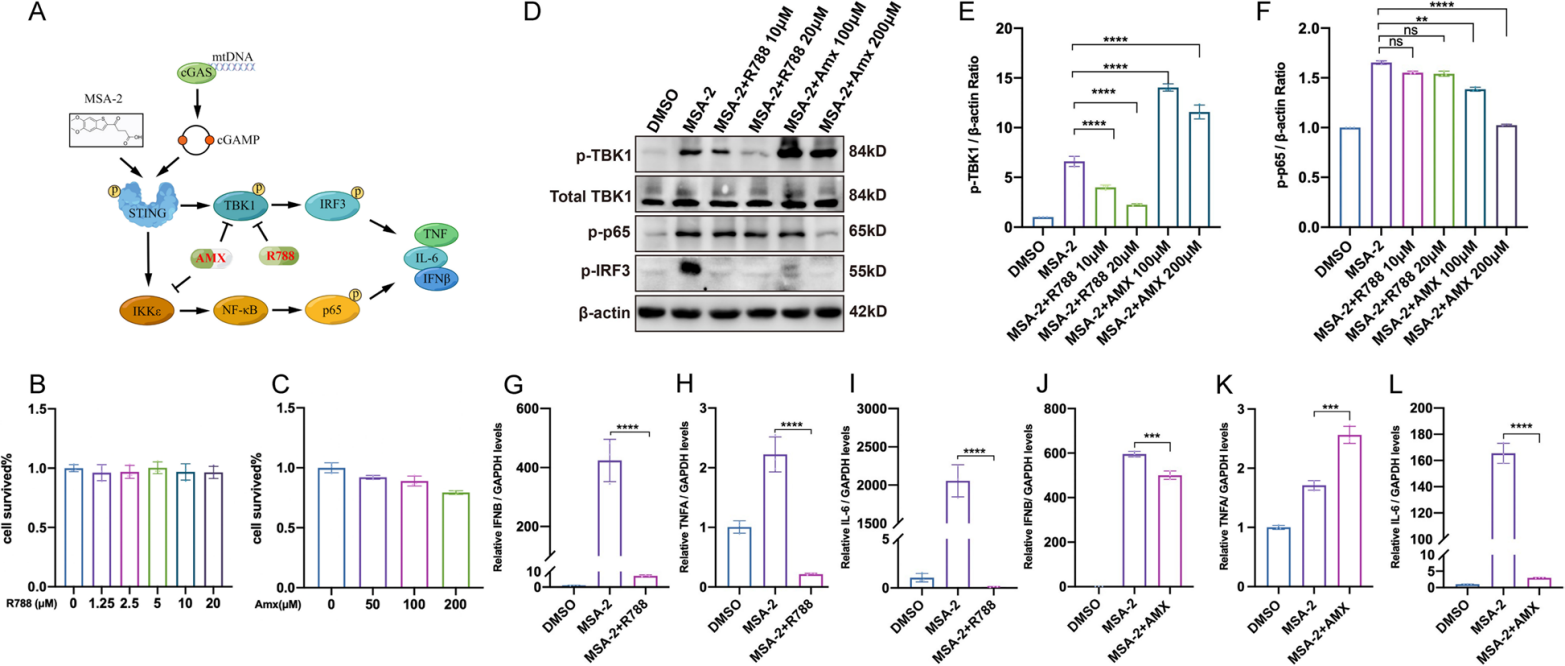
The cGAS-STING pathway activation induced by MSA-2 is suppressed by R788 and AMX. **A** The schematics of signaling interactions between R788/AMX and the cGAS-STING pathway. **B–C** CCK-8 assay showed the cytotoxicities of R788 and AMX with increased dosages. **D** Western blotting analysis of p-p65, p-IRF3, and p-TBK1 from THP-1 cells subjected to MSA-2 stimulation and treated with R788 (10–20 μM) or AMX (100–200 μM). The qualification of p-TBK1 (**E**) and p-p65 (**F**) when normalized with β-actin. **G–L** RT-qPCR showed the relative levels (normalized with GAPDH) of *IFNB*, *TNFA*, and *IL6* from THP-1 cells treated with R788 (10 μM) or AMX (200 μM)

MSA-2 is known as a non-nucleotide STING agonist binding to STING [49]. It stimulates cGAS/STING pathway via the TBK1/IRF3 axis as well as the IKKε/NF-ҡB axis (depicted in Fig. 4A) [49]. CCK-8 results presented slightly increased cytotoxicity in a concentration-dependent manner upon the administration of R788 (1.25-20 μM) or AMX (50–200 μM) (Fig. 4B–C). We then applied the safety concentrations in the following assays accordingly. THP-1 cells were pretreated with R788 or AMX for 4 h before stimulation with MSA-2 (Fig. 1C), and Western blotting revealed that R788 significantly inhibited the levels of phosphorylated TBK1 (p-TBK1) and IRF3 (p-IRF3), with no effect on p65 phosphorylation (p-p65), indicating the specific inhibition on TBK1 within TBK1/IRF3 signaling pathway. In contrast, AMX reduced the levels of both p-p65 and p-IRF3 while further enhancing TBK1 phosphorylation (Fig. 4D–F). This finding was in line with previously published data showing the dual specificity of AMX targeting both TBK1 and IKKε, leading to negative feedback on TBK1 phosphorylation at the Ser172 [50]. Additionally, our WB results showed that the total protein expression levels of p65, IRF3, and IKKε remained unchanged (Fig. S2A). By quantifying the expression of downstream pro-inflammatory genes, we found that induction of *IFNB*, *TNFA*, and *IL-6* could be effectively inhibited by R788 (Fig. 4G–I). Moreover, AMX also effectively reduced the levels of *IFNB* and *IL-6* (Fig. 4J, L), but failed to inhibit *TNFA* expression, probably due to the very marginal response of this cytokine gene in MSA-2 stimulated THP-1 cells (Fig. 4K, Additional file 4: Fig S2B) [51].

### R788 and AMX inhibited cGAS/STING signaling mediated by ALS-related toxic proteins

**Fig. 5:**
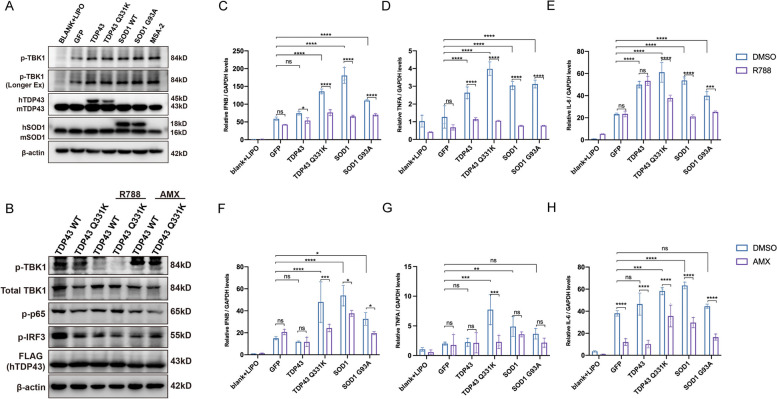
R788 and AMX suppressed cGAS/STING-dependent pro-inflammatory signaling induced by TDP-43/SOD1. **A** Western blotting analyzed the levels of transiently transfected ALS toxic proteins and p-TBK1 in NSC-34 cells. **B** WB analysis of p-TBK1, p-p65, and p-IRF3 in doxycycline-inducible TDP43/Q331K NSC-34 cells. **C–H** RT-qPCR showed the relative levels (normalized with GAPDH) of *IFNB*, *TNFA*, and *IL6* from NSC-34 cells treated with R788 (10 μM) or AMX (200 μM)

Recent research has unveiled cGAS/STING-dependent neuroinflammation in SOD1 and TDP43-related ALS pathogenesis triggered by the mtDNA release [52]. We then conducted a transient transfection assay in NSC-34 cells, using control plasmids (GFP) as well as wild-type (TDP43 WT, SOD1 WT) and mutant (TDP43 Q331K, SOD1 G93A) plasmids (Fig. 1D). We observed that the plasmid transfection per se triggered the TBK1 phosphorylation due to basal cGAS/STING response to the plasmid DNA occurring in the cytosol. In addition, a further increase of p-TBK1 was also observed in the group transfected with SOD1 and TDP43-related plasmids, which confirmed the induction of p-TBK1 by ALS-toxic proteins (Fig. 5A). Furthermore, we applied a doxycycline-inducible system to express TDP43 and Q331K mutant plasmids within NSC-34 cells. The result showed that the p-TBK1 and p-IRF3 were significantly inhibited by R788 in this system (Fig. 5B). We also observed decreased p-p65 and p-IRF3 in AMX-treated groups, where p-TBK1 was intact (Fig. 5B). Furthermore, the total protein expression levels of p65, IRF3, and IKKε remained unchanged (Additional file 4: Fig S2C). This finding was consistent with the reported dual-specificity in inhibiting both TBK1 and IKKε (Fig. 4D) [46]. Moreover, RT-qPCR data demonstrated that R788 and AMX inhibited the expression of the most downstream cGAS/STING effectors we tested in NSC-34 cells overly expressing TDP-43 WT/Q331K or SOD1 WT/G93A (Fig. 5C–F, H). Obvious inhibition of *TNFA* expression upon AMX was only seen in TDP43 Q331K mutant, perhaps due to the insignificant gene expression differences in other transfectants (Fig. 5G). As for the cellular model of another major ALS-related gene, qPCR results showed that R788 inhibited the expression of *IFNB*, *TNFA*, and *IL-6* in NSC-34 cells overexpressing C9orf72 GA50/PA50 [53], while AMX only inhibited the expression of *IFNB* (Additional file 4: Fig S2D-2F) [54–57]. To this end, our findings suggested potential protective effects of R788 and AMX against certain ALS-related neuroinflammation subtypes.

### Phenome-wide MR analysis of ALS prior druggable genes

Because most drugs function through blood circulation, we assessed whether the TBK1-targeted drugs in the blood have beneficial or deleterious effects on other indications. A broader MR screening of 783 non-ALS diseases or traits in the UK Biobank (SAIGE v.0.29) showed no significant association was identified (FDR < 0.05) using the Wald ratio method, indicating these drugs are safe or with few side effects. The summary results are presented in Additional file 4: Fig S3 and Additional file 1: Table S11.

## Discussion

Currently, there is no established therapeutic approach for ALS that is proven to possess high efficacy across major patient populations. By integrating large-scale GWAS data with the druggable genome, we investigated more than 4000 potential gene targets to yield *TNFSF13*, *CD68*, *TNFSF12*, *TBK1*, *RESP18*, *GDF9*, *PTPRN*, and *GPX3* target loci via systematic MR. *TBK1*, *TNFSF12, RESP18*, and *GPX3* were then validated in the colocalization analysis. Further repurposing drug discovery and pharmacological verification analyses were applied to identify *TBK1* as a promising repurposing drug target for ALS treatment. Although we regard *TBK1* as one of the most promising targets for the treatment of ALS, it is essential to emphasize the significant value of *TNFSF12*, *RESP18*, and *GPX3* in ALS drug research as well. Future studies should also focus on the interactions between these genes and others to gain a deeper understanding of their roles in the disease mechanism.

Among the abovementioned genes, *TBK1* encodes a multimeric kinase regulating various biological processes [58] and serves as a known FDA-approved drug target in treating rheumatoid arthritis and multiple sclerosis [59]. Recently, several studies with large human genetic samples have suggested the multi-faceted roles of *TBK1* mutations in the pathogenesis of ALS [60–62]. In genetic results, *TBK1* haploinsufficiency has been found to disrupt motor neuron autophagy, enhance mutated SOD1 protein accumulation, and promote ALS-like phenotypes in transgenic mouse models [63]. Meanwhile, researchers have also highlighted the bi-directional roles of TBK1 in the context of the SOD1^G93A^ ALS mouse model. Gerbino et al. indicated that diminished TBK1 function by point mutation, as well as in SOD1^G93A^/TBK1 cKO (conditional TBK1 deletion in motor neurons) mice, promoted disease progression during the early stages but impeded it in later stages [26]. Similar research revealed that SOD1^G93A^/TBK1^±^ mice carrying TBK1 heterozygous deletion displayed clinical symptoms earlier; however, decelerated disease progression and elevated survival rate [64]. TBK1 acts as an adaptor protein downstream of STING, leading to an augmentation of neuroinflammation via IRF3/IFNB axis [25]. The canonical cGAS/STING signaling is activated upon the presence of foreign DNA in the cytosol, such as that derived from invading bacteria or viruses or leaked self-DNA from the nucleus or mitochondria under pathological conditions [65]. In certain pathological conditions, the excessive TBK1 abundance might be associated with over-activated signaling. Clark et al. observed increased TBK1 protein level paralleled with elevated phosphorylation, which was partially due to intermolecular autophosphorylation [66]. The other study has also reported that overexpression of TBK1 in zebrafish could induce IRF3 phosphorylation [67]. Our MR analysis identified increased TBK1 expression in ALS patients, which may possess over-activated signal transductions. It has been demonstrated that in SOD1/TDP43-associated ALS mice models, the accumulation of toxic SOD1 or TDP43 proteins mislocated in mitochondria was found to trigger the release of mtDNA and RNA:DNA hybrids into the cytosol [52]. These two ALS-related toxic genes and their gain-of-function mutants were found to efficiently stimulate the cGAS/STING pathway through both the TBK1/IRF3 and the IKKε/NF-ҡB axes leading to the subsequent activated expression of inflammatory cytokines. Particularly, in a Prp-TDP-43^Tg/+^ ALS mouse model, the disease was mitigated through genetic deletion or pharmacological inhibition of STING [25]. Therefore, targeting the pro-inflammatory effects of STING activation using specific inhibitors represents a promising therapeutic approach to impede the progression of ALS. A rising number of inhibitors targeting the cGAS/STING pathway have been reported [68].

Compared to innovating novel drugs, repurposing approved drugs for new indications has been demonstrated as a low time–cost and less investment strategy with an improved success rate [69]. For example, metformin has been proven to enhance prognosis in patients with non-diabetic heart failure [70, 71]. In addition, the kinase drug repurposing and novel target discovery have also been widely investigated. For example, imatinib, an FDA-approved ABL kinase inhibitor for chronic myeloid leukemia (CML), was later discovered to effectively treat advanced gastrointestinal stromal tumors by inhibiting KIT kinase [72, 73]. The aurora kinase inhibitor tozasertib has also been successfully used to treat CML-bearing ABL1 kinase mutant (T315I) [74]. Based on Mendelian randomization analysis and previous reports, our drug repurposing analysis on FDA-approved drugs identified the dual-specificity inhibitor AMX known to block TBK1 and IKKε enzymatic activities [75], which is utilized in several ongoing clinic trials [76, 77]. Our in vitro data suggested that AMX efficiently inhibited the IRF3/p65 phosphorylation induced by either cGAS/STING agonist MSA-2 or overexpression of ALS toxic proteins. These effects resulted in the attenuation of downstream pro-inflammatory gene expression (Fig. 4D, J–L).

Furthermore, polypharmacological effects are commonly observed in kinase inhibitor developments due to structural and functional conservation across ATP binding sites, which may have advantageous or detrimental consequences for their clinical applications [78]. Klaeger et al. showed that the FDA-approved MET/VEGFR inhibitor, cabozantinib, effectively inhibited the tyrosine kinase fusion product FLT3-ITD to potentially cure acute myeloid leukemia [78, 79]. As a known SYK inhibitor, R788 has been widely used in autoimmune diseases such as rheumatoid arthritis lymphoma [80] and immune thrombocytopenic purpura [81]. In the current study, we found a significant binding affinity of its active form R406 to TBK1 in serval independent online databases (Kd =  ~ 22 nM) (Lincs, DrugBank, and BindingDB) [47]. The molecular docking results suggested that both R788 and R406 were predicted to be located within the ATP-binding site or ATP-pocket of TBK1, with mostly overlapped interactions shown in the published R406/SYK co-crystal structure (Fig. 3B–D). Our in vitro results showed that R788 effectively inhibited the rise in TBK1/IRF3 phosphorylation levels induced by either cGAS/STING agonist MSA-2 or overexpression of ALS toxic proteins, resulting in the dampened pro-inflammatory gene expression (Fig. 4D, G–I). To this end, the repurposing potential of R788 on TBK1 has been demonstrated feasible by both bio-informative and pharmacological validations. Collectively, the results from in vitro findings and interaction analysis suggested that AMX and R788 might be utilized to suppress cGAS/STING-mediated neuroinflammation, thereby potentially treating ALS subtypes particularly related to overexpressed SOD1, TDP43, and their derivatives, respectively. Overall, by applying druggable genome-wide MR and pharmacological verification in vitro, we affirmed the promising application of AMX and R788 for repurposing uses in ALS. Because AMX and R788 have been approved for marketing by the FDA, their efficacy, safety, and adverse effects have been confirmed, while the expected benefit of conducting clinical trials is high.

In addition, we also identified that targets on *TNFSF12* and *GPX3* had potential protective roles in ALS. As we have discovered that *TNFSF12* overexpression have a potential risk for ALS, MR and colocalization results strongly suggested that this gene could be a promising drug target for ALS. Studies have shown that TNFSF12 and its associated factor, fibroblast growth factor-inducible type 14 (Fn14), is an emerging apoptosis inducer to play an important role in the pathogenesis of muscle atrophy [19], which has also been validated in mouse models and clinical cohorts [82–84]. We searched both ChEMBL and DrugBank Online for drugs that are currently in clinical trials to inhibit *TNFSF12* expression and ultimately identified two drugs: BIIB-023 (ChEMBL ID: CHEMBL2109598) and RG7212 (ChEMBL ID: CHEMBL2109600). BIIB023, a humanized anti-TNFSF12 monoclonal IgG1 antibody, is currently in clinical trials for rheumatoid arthritis and has a favorable safety and tolerability profile [42]. RG7212, a fully humanized IgG1κ monoclonal antibody that attenuates the binding of Fn14 to TNFSF12, has been shown to inhibit tumor growth in multiple in vivo models, including renal, breast, and pancreatic cancers [85, 86]. We speculated that RG7212 and BIIB-023 might improve muscle atrophy in ALS patients through the TNFSF12 pathway, ultimately delaying disease progression. However, due to the uncertain safety and efficacy of RG7212 and BIIB-023, we have not prioritized them as top drug repurposing and have not conducted pharmacological research on them.

*GPX3* gene was found as the unique gene altered in both ALS blood and brain samples. GPX3 is abundantly present in neurons, the brain, blood plasma, and other tissues. It has been reported to reduce oxidative stress by detoxifying hydrogen and soluble lipid peroxides catalyzed by reduced glutathione [21]. A recent study suggested that *GPX3* genetic alteration may play an important role in the pathogenesis of ALS through reactive oxygen species (ROS) [23]. Interestingly, the brain eQTL results suggested that *GPX3* expression reduced the risk of developing ALS, which was contrary to the results in the blood, suggesting that *GPX3* transcriptional and translational regulations might not be coupled in different tissues. We searched both ChEMBL and DrugBank Online with *GPX3* as a target and identified glutathione in documented clinical trial profiles. Glutathione binds to other drugs to make them more soluble for excretion, and it also serves as a cofactor for certain enzymes involved in protein disulfide bond rearrangement and reducing peroxide production [87]. Due to the molecular characteristics and mechanism of action of glutathione, it is better suited as a supplement rather than a unique ALS treatment.

The strengths and innovations of our study include the following. Firstly, our study employed druggable genome MR to investigate potential drug targets and identify repurposed drugs for ALS treatment. Secondly, we meticulously selected multiple tissue datasets with the largest sample size and rigorously conducted verifications using various instrumental variable selection criteria, thereby enhancing the credibility of our findings. Furthermore, our study also validated partial findings from previous research, such as *TBK1* [13], a druggable target identified in our study; however, it was not reported as a prioritized drug target for ALS in other studies [23, 88–90]. Additionally, we have made novel discoveries by identifying new druggable genes in blood samples, brain tissue, and spinal cord (e.g., *TNFSF12*, *TNFSF13*, and *RESP18*), which offer promising avenues for future investigation of ALS drugs.

Our study also has some limitations. Firstly, the results of the MR method may have weak instrument bias, i.e., the degree of phenotypic variation explained by a single genetic instrument is relatively limited, and the sample size needs to be expanded to obtain sufficient precision. Secondly, genetic associations for variants identified through genome-wide association studies tend to be overestimated in the original discovery data set, also known as “winner’s curse bias” [91]. Thirdly, we set a more stringent *r*^2^ value to sufficiently remove the linkage disequilibrium to improve the accuracy of the study, which resulted in only 1 SNP for all our positive results. Thus, our results may suffer from a lack of statistical power but also possess a small intrinsic bias. Furthermore, our data are derived from European populations, which may lead to non-applicability between races. Additionally, our MR analysis was limited to selected known druggable genome eQTLs and ALS GWAS, potentially excluding other potential druggable genes from the analysis. Moreover, our analysis of IVs was based on the set selection criteria, which could introduce selection bias and result in false positive or false negative deviations in this study. It is worth noting that although our research relies on bulk tissue gene expression data, drug treatments may exert their effects specifically on certain cell types or tissues. Therefore, it is crucial to further investigate and address the heterogeneity of tissues and specific cell types. Lastly, it is crucial to underscore that despite the evidence provided by MR and pharmacological validation in cells, successful drug application cannot be guaranteed due to potential disparities in gene expression data between diseased and healthy states. Real-world applications may give rise to unexpected adverse effects or limited clinical benefits.

## Conclusions

Using the druggable genome-wide MR approach, we obtained four potential drug targets for ALS. Repurposing drug discovery found five drugs with repurposing opportunities acting on *TBK1*, *TNFSF12*, and *GPX3*, respectively. After inclusion criteria filtration, we prioritized two FDA-approved TBK1 inhibiting chemicals that act as repurposing drugs for treating certain ALS subtypes and validated their efficacies by in vitro pharmacological analyses. Our research provides new insights into genetic-based drug development for ALS treatment, which will offer potential for successful clinical trials.

### Supplementary Information

**Additional file 1: Table S1-S11. Table S1.** Strengthening the Reporting of Observational Studies in Epidemiology using Mendelian Randomization (STROBE-MR) checklist. **Table S2.** The details of eQTL used in the study. **Table S3.** Primers used in qPCR experiments. **Table S4. **Mendelian Randomization Analysis Results for Blood Gene Expression and ALS Outcomes of SNPs under different criteria. **Table S5.** Mendelian Randomization Analysis Results for Brain Gene Expression and ALS Outcomes of SNPs under different criteria. **Table S6.** Mendelian Randomization Analysis Results for Spinal cord Gene Expression and ALS Outcomes of SNPs under different criteria. **Table S7.** Druggable genes associated with ALS. **Table S8.** Sensitivity analyses by MR-Egger and weighted median methods. **Table S9.** The results of colocalization analysis. **Table S10.** Wald ratio approach supporting the expression of druggable genes significantly associated with ALS risk. **Table S11.** The phenome-wide MR results of blood TBK1 in IVW or Wald ratio method.

**Additional file 2. **Supplementary methods of confirmatory eQTLs data and cellular thermal shift assay.

**Additional file 3. **Supplementary result of confirmatory MR analysis.

**Additional file 4: Fig. S1-S3. Fig. S1.** The molecular docking results of AMX with TBK1. And the cellular thermal shift assay results for TBK1 and AMX/R788. **Fig. S2.** Results for total protein expression levels of p65, IRF3 and IKKε. And the RT-qPCR results for *IFNB*, *TNFA*, and *IL-6 *related to C9orf72. **Fig. S3.** Results of the phenome-wide MR association analysis on TBK1 expression for clinical outcomes in the UK Biobank.

## Data Availability

All data is obtained from open access and the specific paths are provided in this article.

## Abbreviations

ALS: Amyotrophic lateral sclerosis
AMX: Amlexanox
CETSA: Cellular thermal shift assay
cGAS: Cyclic GMP-AMP synthase
*cis*-eQTL: Cis-Expression quantitative trait loci
IFN: Type I interferon
IVW: Inverse-variance weighted
MR: Mendelian randomization
PD: Parkinson’s disease
PP3: Posterior probability for H3
p-TBK1: Phosphorylated TBK1
R788: Fostamatinib
SOD1: Superoxide dismutase 1
STING: Stimulator of interferon genes
TBK1: TANK-binding kinase-1
TDP-43: TAR DNA-binding protein
TSS: Transcription start site
