# Supplementary material for: TBK1, a prioritized drug repurposing target for amyotrophic lateral sclerosis: evidence from druggable genome Mendelian randomization and pharmacological verification in vitro
Source: BMC Med. 2024 Mar 5;22:96. doi: 10.1186/s12916-024-03314-1 (PMC10916235; doi:10.1186/s12916-024-03314-1)
Supplement: Supplementary file 2 — Additional file 2. Supplementary methods of confirmatory eQTLs data and cellular thermal shift assay. [file 12916_2024_3314_MOESM2_ESM.docx]

**Supplementary Method**

**Confirmatory eQTLs data**

To confirm whether the genes identified in the discovery MR analysis were associated with ALS risk, we conducted further MR analyses using different MR parameters and eQTL data. In the selection of IVs, we employed additional various P-values (1e-05 and 5e-08) to investigate the group of genes. Furthermore, SNPs within ±1MB from the transcription start site (TSS) that met the FDR<0.05 criterion were included to validate our findings. Further, we conducted another set of two-sample MR with the same MR parameters (SNPs within the ±100kb from TSS, FDR<0.05) to investigate the relationship between blood and brain gene eQTL and ALS. We obtained eQTL datasets for blood, brain cortex, and brain frontal cortex from the GTEx version 8 dataset. A summary of the complete eQTL data for the relevant tissues can be downloaded from the GTEx portal (<https://www.gtexportal.org/home/datasets>). Additionally, we utilized eQTL data from the spinal cord for further validation, spanning the cervical, thoracic, and lumbar regions. These spinal cord data were obtained from a recently published dataset by the NYGC ALS Consortium, comprising 216 cervical spinal cord samples, 197 lumbar spinal cord samples, and 68 thoracic spinal cord samples. All the details of the eQTL data are presented in **Supplementary Table 2**.

**Cellular thermal shift assay (CETSA)**

In a nutshell, we collected approximately 1*10^7^ NSC-34 cells and suspended them in 1 mL of PBS with 1% protease and phosphatase inhibitor single-use cocktails. To break down the cells, we subjected the suspension to five cycles of freezing and thawing using liquid nitrogen. After that, we centrifuged the cell lysate at 14,000g for 10 minutes at 4°C, collected the supernatant, and split it into two equal portions. One portion was treated with R788 (100 μM), while the other received an equal volume of DMSO. Both portions were incubated at 26°C for 1 hour. Next, we divided the R788 and DMSO-treated cell lysates into seven equal parts and heated them for 3 minutes at temperatures ranging from 37 to 60°C. Once again, we centrifuged the samples at 14,000g for 10 minutes (4°C) to collect the soluble fraction. We used western blotting to evaluate the thermal stability of the target protein. Furthermore, we repeated the experiment using AMX (1 mM) under the same experimental conditions.
