# Supplementary material for: TBK1, a prioritized drug repurposing target for amyotrophic lateral sclerosis: evidence from druggable genome Mendelian randomization and pharmacological verification in vitro
Source: BMC Med. 2024 Mar 5;22:96. doi: 10.1186/s12916-024-03314-1 (PMC10916235; doi:10.1186/s12916-024-03314-1)
Supplement: Supplementary file 3 — Additional file 3. Supplementary result of confirmatory MR analysis. [file 12916_2024_3314_MOESM3_ESM.docx]

**Supplementary Result**

**Genetic** **Instrument Variants Selection**

In the confirmatory phase using different MR parameters, we included a series of SNPs as IVs. For SNPs located within ±100kb of the TSS, meeting the criteria of P<5e-08 and FDR<0.05, we utilized 1239 SNPs for 1057 blood-druggable genes and 621 SNPs for 604 brain-druggable genes as instrumental variables. When considering SNPs within ±100kb of the TSS with P<1e-05 and FDR<0.05, we employed a total of 1332 SNPs for 1121 blood-druggable genes and 758 SNPs for 719 brain-druggable genes as IVs. Lastly, when incorporating SNPs within ±1Mb of the TSS with an FDR<0.05, we employed a total of 1681 SNPs for 1227 blood-druggable genes and 1036 SNPs for 938 brain-druggable genes as IVs.

In the confirmatory phase using different eQTL data, we employed a total of 655 SNPs for 635 blood-druggable genes, 257 SNPs exclusively for 257 frontal brain cortex-druggable genes, and 766 SNPs allocated to 765 whole brain cortex-druggable genes as IVs. Additionally, we utilized 431 SNPs for 425 cervical spinal cord-druggable genes, 373 SNPs for 373 lumbar spinal cord-druggable genes, and 23 SNPs for 23 thoracic spinal cord-druggable genes.

**MR Analysis Between Gene Expression and ALS Outcomes**

In the confirmatory phase, we have consistently observed an association between the mentioned genes in the blood (*TNFSF13*, *CD68*, *TNFSF12* and *TBK1*) and brain (*RESP18*, *GDF9*, and *GPX3*) and ALS under different IVs selection criteria, with their effects consistently in the same direction as mentioned above. The MR analysis results for blood gene expression and ALS outcomes can be found in **Supplementary Table 4**, while the MR analysis results for brain gene expression and ALS outcomes are available in **Supplementary Table 5**. The list of potentially druggable genes is presented in **Supplementary Table 7**, and the summary of MR results from the discovery phase is depicted in **Figure 2**.

In the confirmatory phase using different eQTL data, we validated an increased ALS risk associated with *TBK1* and *TNFSF12* in the blood. Furthermore, in data from the whole brain cortex, frontal cortex, and cervical spinal cord, we also confirmed an association between the *RESP18* gene and ALS risk. The MR analysis results of blood and brain gene expression and ALS outcomes are presented in **Supplementary Table 4**, while the MR analysis results of spinal cord gene expression and ALS outcomes are shown in **Supplementary Table 6**.
