# Supplementary material for: TBK1, a prioritized drug repurposing target for amyotrophic lateral sclerosis: evidence from druggable genome Mendelian randomization and pharmacological verification in vitro
Source: BMC Med. 2024 Mar 5;22:96. doi: 10.1186/s12916-024-03314-1 (PMC10916235; doi:10.1186/s12916-024-03314-1)
Supplement: Supplementary file 4 — Additional file 4: Fig. S1-S3. Fig. S1. The molecular docking results of AMX with TBK1. And the cellular thermal shift assay results for TBK1 and AMX/R788. Fig. S2. Results for total protein expression levels of p65, IRF3 and IKKε. And the RT-qPCR results for IFNB, TNFA, and IL-6 related to C9orf72. Fig. S3. Results of the phenome-wide MR association analysis on TBK1 expression for clinical outcomes in the UK Biobank. [file 12916_2024_3314_MOESM4_ESM.docx]

**Supplementary Figures**

| **Supplementary Figure 1.** The molecular docking results of AMX with TBK1. And the cellular thermal shift assay results for TBK1 and AMX/R788. |
| --- |
| **Supplementary Figure 2.** Results for total protein expression levels of p65, IRF3 and IKKε. And the RT-qPCR results for *IFNB*, *TNFA*, and *IL-6* related to C9orf72.  **Supplementary Figure 3.** Results of the phenome-wide MR association analysis on TBK1 expression for clinical outcomes in the UK Biobank. |


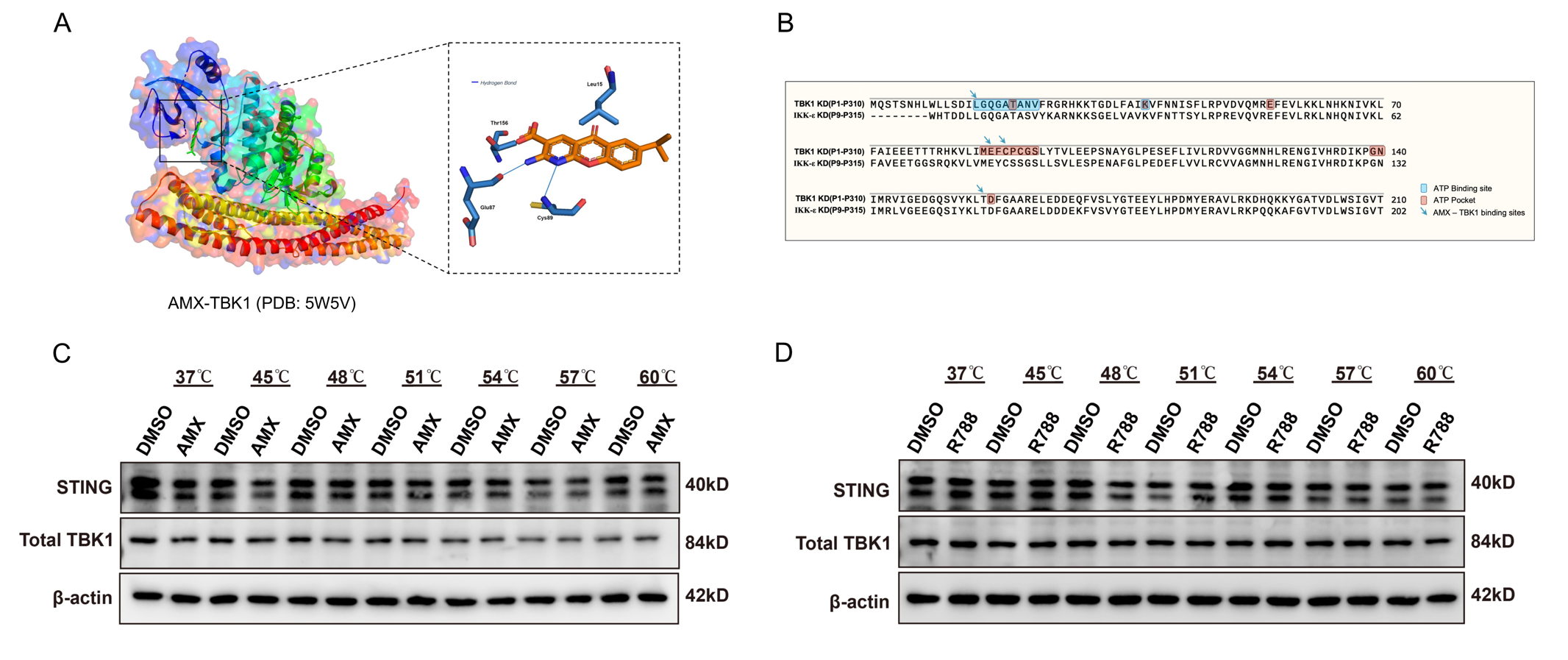


**Supplementary Figure 1.** (A) The global (left) and detailed (right) schematics illustrate the interaction between AMX and TBK1 by co-crystal structure (PDB: 5W5V) (hydrogen bonds are depicted by solid blue line). (B) The amino acid sequence comparison within kinase domains of TBK1 and IKKε. (C-D) The impact of AMX/R788 on the thermal stability of TBK1 in NSC-34 cell lysates was investigated through cellular thermal shift assay


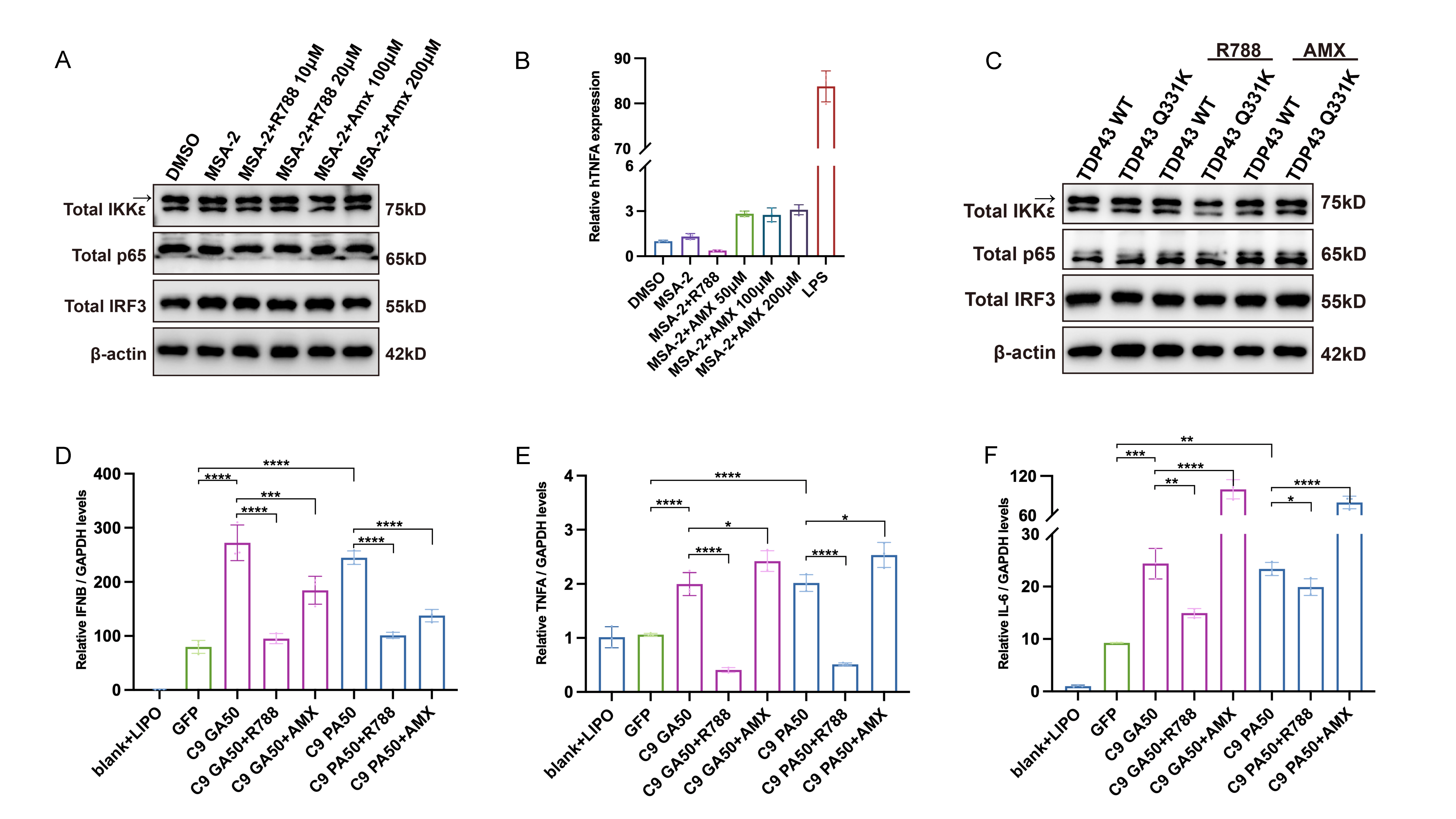


**Supplementary Figure 2.** (A) Western blotting analysis for total protein expression levels of p65, IRF3 and IKKε from THP-1 cells subjected to MSA-2 stimulation and treated with R788 (10-20μM) or AMX (100-200μM). (B) RT-qPCR showed the relative levels (normalized with GAPDH) of *TNFA* from THP-1 cells treated with R788 (10μM), AMX (200μM), or LPS(40 ng/mL). (C) Western blotting analysis for total protein expression levels of p65, IRF3 and IKKε in doxycycline-inducible TDP43/Q331K NSC-34 cells.(D-F) RT-qPCR showed the relative levels (normalized with GAPDH) of *IFNB,* *TNFA*, and *IL-6* from NSC-34 cells treated with R788 (10μM) or AMX (200μM).


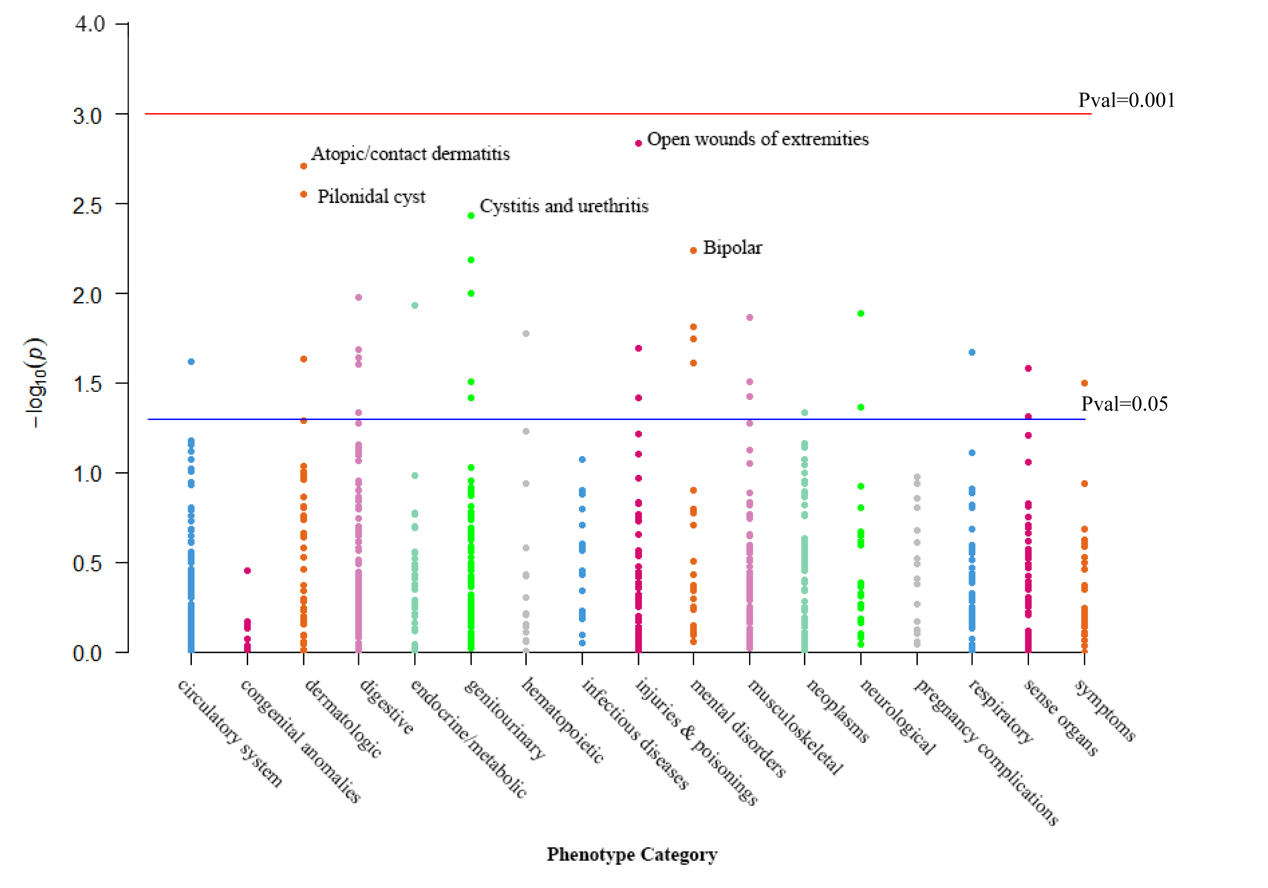


**Supplementary Figure 3.** Results of the phenome-wide MR association analysis on TBK1 expression for clinical outcomes in the UK Biobank. The horizontal axis represents the phenotype category taken from the UK biobank, and the vertical axis corresponds to the logarithms of the *P* values derived from the phenome-wide MR association analyses. The red line represents the *P* value is 0.001, and the blue line represents the *P* value < 0.05.
